# A Unimodal Model for Double Observer Distance Sampling Surveys

**DOI:** 10.1371/journal.pone.0136403

**Published:** 2015-08-28

**Authors:** Earl F. Becker, Aaron M. Christ

**Affiliations:** Alaska Department of Fish and Game, Anchorage, Alaska, United States of America; University of Missouri Kansas City, UNITED STATES

## Abstract

Distance sampling is a widely used method to estimate animal population size. Most distance sampling models utilize a monotonically decreasing detection function such as a half-normal. Recent advances in distance sampling modeling allow for the incorporation of covariates into the distance model, and the elimination of the assumption of perfect detection at some fixed distance (usually the transect line) with the use of double-observer models. The assumption of full observer independence in the double-observer model is problematic, but can be addressed by using the point independence assumption which assumes there is one distance, the apex of the detection function, where the 2 observers are assumed independent. Aerially collected distance sampling data can have a unimodal shape and have been successfully modeled with a gamma detection function. Covariates in gamma detection models cause the apex of detection to shift depending upon covariate levels, making this model incompatible with the point independence assumption when using double-observer data. This paper reports a unimodal detection model based on a two-piece normal distribution that allows covariates, has only one apex, and is consistent with the point independence assumption when double-observer data are utilized. An aerial line-transect survey of black bears in Alaska illustrate how this method can be applied.

## Introduction

Animal population size is an important parameter for many wildlife and ecological problems. Distance sampling is a widely used method to estimate animal population size [1]. Conventional distance sampling (CDS) models animal detection as function of the perpendicular distance between the animal and transect to obtain a population estimate. In most applications, there are additional variables that affect an animal’s detection probability [2–4] and these variables can be used for modeling animal detection. When appreciable variation in animal detection is present, multiple covariate distance sampling (MCDS) models are superior to CDS models if the appropriate covariates are measured [5]. CDS and MCDS models make the important assumption that animal detection is perfect at the apex of detection, usually on the transect line (distance = 0). If this assumption is true, Burnham et al. [6] show that CDS models under mild assumptions are robust to biases associated with unmodeled heterogeneity, a condition Burnham et al. [7] labeled “pooling robust”. By extension, MCDS models would also be pooling robust if the assumption of perfect detection at the apex were true.

In many cases the perfect detection assumption is unrealistic or suspect. In such cases the collection of double-observer data can be used to remove this assumption. Double-observer data consists of 2 observers trying to detect animals independently of one another. When one observer detects an animal, it is essentially “marked” for the other observer to either detect (recapture) or miss. The incorporation of double-observer data into distance modeling (Mark-Recapture Distance Sampling models, MRDS models) eliminates the assumption of perfect detection at a distance associated with the detection apex and allows this parameter to be estimated [8–11] and even vary due to other variables [11]. The initial MRDS models [8–11] assume independent animal sightings between the observers over all distances, which Borchers et al. [12] call the “full independence” (FI) assumption. While there are several ways to estimate MRDS models under this assumption, a common method is to use a Huggins type [13] mark-recapture model with distance as a covariate [14]. The property of “pooling-robustness” does not hold for MRDS models [6]. Like mark-recapture models, unmodeled heterogeneity in MRDS models is a source of bias [12]. Covariates can reduce the unmodeled heterogeneity but are unlikely to account for all the sources of heterogeneity [15].

Laake [16] analyzed distance data on a known population of 150 wooden stakes, the best CDS estimate was 81% (121) of the true value, while the MRDS model using the FI assumption was 80% (120) of the true value. Additionally, analysis of crabeater seal (*Lobodon carcinophaga*) [12,14] and feral horse (*Equus caballus*) [15] datasets with a MRDS model using the FI assumption resulted in estimates that were considerably lower than those obtained with MCDS models that assume perfect detection on the transect line (the location of maximum detection for these data). These results do not follow the expected pattern of MRDS population estimates being larger than CDS and MCDS estimates. These results can be caused by statistical dependence in the data, even though the 2 observers collected the data independently. The dependence can easily be a function of distance or animal activity. Increased distance to the animal will cause more and more animals to be missed. If at far distances only the most visible animals are detected then they may be more likely to be detected by both observers since they are more visible than the average animal at that distance. This occurs because missing variables explaining detection have not been accounted for; this is unmodeled heterogeneity. In the above case, an animal standing on a ridge top with no ground cover or vegetation in the background (sky-lined), would be very visible at far distances to both observers; the inadequate modeling of the sighting conditions would cause unmodeled heterogeneity. The effect of vegetation obstruction may increase with distance, resulting in the situation described above. Laake et al. [15] document a divergence in detection probabilities over distance obtained from a MCDS model and the mark-recapture probabilities in a feral horse dataset. The higher mark-recapture probabilities at far distances are consistent with unmodeled heterogeneity causing some dependency in the sighting data. Laake et al. [15] state: “it is important to understand that independence is not solely achieved by the observers being unaware of each other.”

There are several ways to deal with this dependence issue. Buckland et al. [17] used a limited independence model that simultaneously modeled the distance data, the mark-recapture data and the dependence between the 2 observers as a function of distance as well as other covariates. Conn et al. [18] modeled observer detections as arising from a probit-Bernoulli model with the correlation between the observers being a function of distance. Laake [16] presented a less restrictive independence assumption that only assumed independence of observer observations at the distance associated with the apex of detection. Borchers et al. [12] denoted these as “point independence” (PI) assumption models. MRDS models that use a point independence assumption fit a MCDS model and use a Mark-recapture model to estimate maximum detection at the MCDS detection apex, this is then used to adjust the height of the MCDS model [12,16]. Borchers et al. [12] document the mathematical details of a MRDS model under the PI assumption.

Line transect surveys are an excellent tool for obtaining population estimates for large geographic areas. In terrestrial study areas, aircraft are often used to fly transects over large study areas. Assuming perfect detection at some distance from a fast-moving aircraft is unrealistic and requires the use of a MRDS model to estimate animal detection. Due to costs, both observers are usually on the same aircraft which makes it more likely that unmodeled heterogeneity of the sighting conditions will occur. Under these conditions a MRDS model using the PI assumption will be less biased than one that relies on the FI assumption [12, 16]. Large terrestrial study areas often contain mountainous terrain over which it is unsafe to fly straight-line transects; however, these areas can safely be flown using contour transects [19–20]. For contour transects, distance is measured as the closest distance between the transect and the sighted animal using GPS locations [19–21]. Becker and Quang [20] encountered unimodal detection data of brown bears sighted from transects flown with small fixed-wing aircraft. The main reason for this unimodal detection shape is that detection is degraded close to the aircraft due to less time available for searching caused by the plane’s speed and fewer sighting angles around vegetative obstructions, whereas at moderate distances the increased sighting angles and viewing time improve detection despite the negative effect of increased distance. A more detailed explanation is available on pages 209 and 220 of Becker and Quang [20]. They used a unimodal detection function coupled with double-count data modeled under the FI assumption to obtain a population estimate. They also modeled the scale parameter as a function of covariates similar to Marques and Buckland [4]. In the gamma detection model, the addition of covariates results in a different detection apex for each covariate level [20]. The gamma detection function when coupled with double-count data is inconsistent with a MRDS model utilizing the PI assumption, since the covariates cause multiple apexes at various distances and under the PI assumption only 1 distance is assumed to have point independence [12].

The purpose of this paper is to implement a MRDS model for unimodal detection data that does not require independent observations over all distances between the 2 observers. Our solution will involve developing a new detection model that will allow us to implement Borchers et al. [12] MRDS model with the PI assumption. We propose a new detection model that has a unimodal shape with only one detection apex, even when multiple covariates are used to model detection. Additionally, a unimodal model for the double-observer (mark-recapture) data will be presented. A MRDS model using the PI assumption allows for less biased and more realistic population estimates from line transect data which have a unimodal detection shape and avoids the unrealistic assumption of perfect detection [12,16]. If there is no unmodeled heterogeneity then the population estimates are unbiased [12]. As an example, these models will be applied to double-observer, aerial line transect survey of black bears (*Ursus americanus*).

## Methods

Borchers et al. [12] partition the likelihood for MRDS data into 4 components: a distance component $\left(L_{y|z}\left({\underline{\theta }}_{y|z}\right)\right)$, a mark-recapture component $\left(L_{\omega }\left({\underline{\theta }}_{\omega }\right)\right)$, a binomial component $\left(L_{n•}\left({N_{c},\underline{\theta },\underline{\phi }}_{\underline{z}}\right)\right)$, and a covariate component $\left(L_{z}\left(\phi_{z},\underline{\theta }\right)\right)$ Using their partial likelihood approach, estimates of θ_,(θ_={θ_,y|zθ_ω}) can be obtained from $\left(L_{y|z}\left({\underline{\theta }}_{y|z}\right)\right)$, $\left(L_{\omega }\left({\underline{\theta }}_{\omega }\right)\right)$, and a Horvitz-Thompson like estimator is used to obtain the population estimate. Our focus is on aerial surveys which often have blind strip of width (*w*_*b*_), we use the notation of Borchers et al. [12] with the lower limit denoted by *w*_*b*_ throughout this paper.

### Distance Data

Assume random transect placement and truncation of the distance data at *w*. Let *y* represent the closest distance between the transect and the sighted animal, $\underline{z}$ represent a vector of covariates that affect the detection probability, and *i* index the sighted animal (*i* = 1,2,…*n*). Define $p_{•}\left(y_{i},{\underline{z}}_{i}\right)$ as the probability that an animal at distance *y*_*i*_ with covariate vector ${\underline{z}}_{i}$ is detected by at least one observer in the (*w*_*b*_,*w*) strip. Denote the pdf of *y* as $\pi \left(y\right)=\frac{1}{\left(w-w_{b}\right)}$.

Borchers et al. [12] give the general form of the distance likelihood as:

$$L_{y|z}\left({\underline{\theta }}_{y|z}\right)=\prod_{i=1}^{n}\frac{p_{•}\left(y_{i},{\underline{z}}_{i}\right)\pi \left(y\right)}{p_{•}\left({\underline{z}}_{i}\right)},$$ (1)

where

$$p_{•}\left({\underline{z}}_{i}\right)=\int_{w_{b}}^{w}p_{•}\left(y_{i},{\underline{z}}_{i}\right)\pi \left(y\right)dy,$$ (2)

is the probability that at least one observer detects animal *i*.

Our interest is to develop a MRDS model that is consistent with the PI assumption of independent sighting data between observers only at some distance *y* = y*. This requires a unimodal detection function that also allows the use of detection covariates. The two-piece normal distribution [22], also known as a split-normal distribution, is a simple distribution that meets this requirement. The two-piece normal distribution is essentially 2 half-normal distributions that share a common mode (*μ*) but have different variances. Using the terminology of Marques and Buckland [4] the key function is:

$$p_{•}\left(y_{i},{\underline{z}}_{i}\right)=\{\begin{array}{l} \exp\left[-{\left(\frac{\left(y_{i}-\mu \right)}{\sqrt{2}\sigma_{1i}}\right)}^{2}\right]{\;\text{for w}}_{b}\leq y_{i}<\mu \\ \;\exp\left[-{\left(\frac{\left(y_{i}-\mu \right)}{\sqrt{2}\sigma_{2i}}\right)}^{2}\right]\;\text{for}\;y_{i}\geq \mu , \end{array}$$ (3)

where *μ* denotes the mode of the detection distribution and *μ* = y* under the PI assumption.

Assuming the covariates, ${\underline{z}}_{i}$, affect detection via the scale parameters (*σ*_1*i*_,*σ*_2*i*_) [4],

$$\sigma_{1i}=\exp\left(\beta_{0}+\sum_{k_{1}}\beta_{k_{1}}{\underline{z}}_{k_{1}\text{i}}+\sum_{k_{2}}I\left(w_{b}\leq y_{i}<\mu \right)\beta_{k_{2}}{\underline{z}}_{k_{2}\text{i}}\right),\;\text{and}$$ (4)

$$\sigma_{2i}=\exp\left(\beta_{0}+\sum_{k_{1}}\beta_{k_{1}}{\underline{z}}_{k_{1}\text{i}}+\sum_{k_{3}}I\left(y_{i}\geq \mu \right)\beta_{k_{3}}{\underline{z}}_{k_{3}\text{i}}\right).$$ (5)

The covariates that affect the scale of both half-normal curves are modeled by the $\sum_{k_{1}}\beta_{k_{1}}{\underline{z}}_{k_{1}i}$ terms, the covariates that affect only *σ*_1*i*_ are modeled by the $\sum_{k_{2}}\beta_{k_{2}}{\underline{z}}_{k_{2}i}$ terms, and the covariates that affect only *σ*_2*i*_ are modeled by the $\sum_{k_{3}}\beta_{k_{3}}{\underline{z}}_{k_{3}i}$ terms. The total number of covariates in the model is *k*, (*k* = *k*_1_+*k*_2_+*k*_3_). The *I*( ) notation is a standard indicator function that is 1 when the condition in the parentheses is true and 0 otherwise, the purpose of this notation is to make explicit which scale parameters are being modelled.

MCDS parameter estimates are obtained by maximizing the log-likelihood equation:

$$\begin{array}{l} l_{y|z}\left({\hat{\underline{\theta }}}_{y|z}\right)=\sum_{i=1}^{n}\left[\ln\left(p_{•}\left(y_{i},{\underline{\text{z}}}_{i}\right)\right)+\ln\left(\pi \left(y\right)\right)-\ln\left(p_{•}\left({\underline{\text{z}}}_{i}\right)\right)\right]\\ =\sum_{i=1}^{n}\left[-{\left(\frac{y_{i}-\hat{\mu }}{\sqrt{2}\left[I\left(w_{b}\leq y_{i}<\hat{\mu }\right){\hat{\sigma }}_{1i}+I\left(y_{i}\geq \hat{\mu }\right){\hat{\sigma }}_{2i}\right]}\right)}^{2}\right]\\ -\sum_{i=1}^{n}\ln\left(\int_{y=w_{b}}^{\hat{\mu }}\exp\left[-{\left(\frac{y_{i}-\hat{\mu }}{\sqrt{2}{\hat{\sigma }}_{1i}}\right)}^{2}\right]dy\right)-\sum_{i=1}^{n}\ln\left(\int_{y=\hat{\mu }}^{w}\exp\left[-{\left(\frac{y_{i}-\hat{\mu }}{\sqrt{2}{\hat{\sigma }}_{2i}}\right)}^{2}\right]dy\right)\underset{•}{} \end{array}$$ (6)

Numerical methods for maximizing Eq (6) above require initial starting values for the parameters. We follow Ramsey et al. [23] to obtain initial starting values for the β^k's, and use the apex of a kernel estimator [24] for the initial starting value of $\hat{\mu }$.

### Double-Observer Data

Our focus is the implementation of a point independence MRDS model, which will utilize the results of a two-piece normal distance model in order to obtain a population estimate. Following Borchers et al. [12] the mark-recapture likelihood is:

$$L_{\omega }\left({\underline{\theta }}_{\omega }\right)=\prod_{i=1}^{n}\frac{\mathrm{Pr}\left(\omega_{i}|y_{i},\underline{z}\right)}{p_{•}\left(y_{i},{\underline{z}}_{i}\right)}.$$ (7)

Here *ω*_*i*_ denotes the encounter history of the *i* ^th^ animal (*ω*_*i*_ ∈ {(1,0),(01),(1,1)}); the first number in the history indexes whether the animal was seen by the 1^st^ observer (0 = no, 1 = yes) and the second number indexes whether the animal was seen by the 2^nd^ observer.

Borchers et al. [12] denote the degree of dependence between the 2 observers as $\delta \left(y,\underline{z}\right)$, *σ*_12_ as the covariance between the 2 observers’ detection probabilities that is induced by excluding covariates $\underline{u}$ from the model, and $p_{j}\left(y,\underline{z}\right)={\text{E}}_{u}\left[p_{j}\left(y,\underline{z},\underline{u}\right)\right]$ for observer *j*, *j* = 1, 2;

$$\delta \left(y,\underline{z}\right)=1+\left(\frac{\sigma_{12}\left(y,\underline{z}\right)}{p_{1}\left(y,\underline{z}\right)p_{2}\left(y,\underline{z}\right)}\right),$$ (8)

$$\sigma_{12}\left(y,\underline{z}\right)=Cov_{\underline{u}}\left[p_{1}\left(y,\underline{\text{z}},\underline{u}\right),p_{2}\left(y,\underline{\text{z}},\underline{u}\right)\right].$$ (9)

The calculation of $p_{•}\left(y_{i},{\underline{z}}_{i}\right)$ requires knowledge of $\delta \left(y_{i},{\underline{z}}_{i}\right)$ which cannot be obtained from the capture history [12],

$$p_{•}\left(y_{i},{\underline{z}}_{i}\right)=p_{1}\left(y_{i},{\underline{z}}_{i}\right)+p_{2}\left(y_{i},{\underline{z}}_{i}\right)-p_{1}\left(y_{i},{\underline{z}}_{i}\right)p_{2}\left(y_{i},{\underline{z}}_{i}\right)\delta \left(y_{i},{\underline{z}}_{i}\right).$$ (10)

They show that using conditional probabilities under the PI assumption allows for the estimation of $p_{•}\left(y_{i},{\underline{z}}_{i}\right)$. Without making any independence assumptions:

$$p_{•}^{c}\left(y_{i},{\underline{z}}_{i}\right)=p_{1|2}\left(y_{i},{\underline{z}}_{i}\right)+p_{2|1}\left(y_{i},{\underline{z}}_{i}\right)-p_{1|2}\left(y_{i},{\underline{z}}_{i}\right)p_{2|1}\left(y_{i},{\underline{z}}_{i}\right),$$ (11)

$$p_{•}^{c}\left(y_{i},{\underline{z}}_{i}\right)=p_{•}\left(y_{i},{\underline{z}}_{i}\right)\delta \left(y_{i},{\underline{z}}_{i}\right).$$ (12)

Assuming point independence (PI) at y*, we assume $\delta \left(y*,{\underline{z}}_{i}\right)=1$, so

$$p_{•}^{c}\left(y*,{\underline{z}}_{i}\right)=p_{•}\left(y*,{\underline{z}}_{i}\right)\delta \left(y{*}_{i},{\underline{z}}_{i}\right)=p_{•}\left(y*,{\underline{z}}_{i}\right).$$ (13)

Their approach to fitting a point independence MRDS model requires a logistic model that contains different observers (1 and 2), and other possible covariates to obtain estimates of ${\hat{p}}_{1|2}\left(y_{i},{\underline{z}}_{i}\right)$, and ${\hat{p}}_{2|1}\left(y_{i},{\underline{z}}_{i}\right)$. A linear form of distance (*y*) was a potential explanatory variable in their logistic models. In addition to the linear form of distance as a potential covariate, we also wanted to consider other forms that could mimic the general shape of the two-piece normal detection model. We used a polynomial spline [25] on the distance variable as a possible covariate to accomplish this. We placed no restrictions on the apex location of the spline fit. Depending upon the nature of the unmodeled heterogeneity and other variables being considered in the model, distance may not be an important covariate [15] or have a unimodal shape. Our MRDS model uses a two-piece normal detection function to model the distance data, so we assume point independence at the detection apex $y*=\hat{\mu }$ and obtain ${\hat{p}}_{1|2}\left({\hat{\mu }}_{i},{\underline{z}}_{i}\right)$, and ${\hat{p}}_{2|1}\left(\hat{\mu },{\underline{z}}_{i}\right)$ from our logistic model to calculate ${\hat{p}}_{•}\left(\hat{\mu },{\underline{z}}_{i}\right)$,

$${\hat{p}}_{•}\left(\hat{\mu },{\underline{z}}_{i}\right)={\hat{p}}_{1|2}\left(\hat{\mu },{\underline{z}}_{i}\right)+{\hat{p}}_{2|1}\left(\hat{\mu },{\underline{z}}_{i}\right)-{\hat{p}}_{1|2}\left(\hat{\mu },{\underline{z}}_{i}\right){\hat{p}}_{2|1}\left(\hat{\mu },{\underline{z}}_{i}\right).$$ (14)

### Population Estimation

Following Borchers et al. [12], let *N*_*c*_ be the number of animals in the covered region, which is estimated by using a Horvitz-Thompson like estimator:

$${\hat{N}}_{c}=\sum_{i=1}^{n}\frac{s_{i}}{{\hat{\pi }}_{i}},$$ (15)

where *s*_*i*_ denotes the size of the *i* ^th^ animal group, and ${\hat{\pi }}_{i}$ denotes the estimated inclusion probability of the *i* ^th^ group and is estimated by:

$${\hat{\pi }}_{i}={\hat{p}}_{•}\left(\hat{\mu },{\underline{z}}_{i}\right){\hat{p}}_{•}\left({\underline{z}}_{i}\right)$$ (16)

[12]. The ${\hat{p}}_{•}\left(\hat{\mu },{\underline{z}}_{i}\right)$ term is calculated from the mark-recapture data (Eq (14)) and the ${\hat{p}}_{•}\left({\underline{z}}_{i}\right)$ term is calculated from the distance data (Eq (2)). Transects were randomly placed so inferences about animal density and population size can be made for the entire study area.

Laake and Borchers [14] have shown that averaging the ${\hat{p}}_{•}\left(\hat{\mu },{\underline{z}}_{i}\right)$’s over the observed covariates is also valid and can be used to calculate the estimated inclusion probability. We denote this average as ${\hat{p}}_{•}\left(\hat{\mu }\right)$, and followed equation 6.50 of Laake and Borchers [14]:

p^•(μ^)=∑i=1np^•(μ^,z_i)E^(p^•(z_i))∑i=1n1E^(p^•(z_i)), (17)

where:

$$\hat{E}\left({\hat{p}}_{•}\left({\underline{z}}_{i}\right)\right)=\int_{w_{b}}^{w}{\hat{p}}_{•}\left(y,{\underline{z}}_{i}\right)\pi \left(y\right)dy.$$ (18)

The estimated inclusion probabilities are now calculated by:

$${\hat{\pi }}_{i}={\hat{p}}_{•}\left(\hat{\mu }\right){\hat{p}}_{•}\left({\underline{z}}_{i}\right).$$ (19)

The variance can be obtained following Borchers et al. [12] or by using a transect-based bootstrap method. We used the lowest AIC [1] to determine the best MCDS model and the best logistic model. Marques and Buckland [4] report that estimators based on estimated Horvitz-Thompson inclusion probabilities can have substantial bias if many of the estimated inclusion probabilities are small. Marques and Buckland [5] recommend restricting model selection to models whose estimated inclusion probabilities are all greater than 0.1 and less than 5% of them are below 0.2; we excluded models that did not meet these criteria from our AIC selection process. Assessing the fit of the MCDS model includes a Goodness-of-Fit test [1], q-q plots, and a Kolmogorov-Smirnov test [6]. Model fit for the logistic model and the MRDS model follow Borchers et al. [12].

### Application to a South-Central Alaska Black Bear Population

Data collection involved aerial observation of black bears (*Ursus americanus*), for which no agency field permit or approval was required. All Federal Aviation Administration regulations were followed in collecting the data. No lands were accessed in the data collection, nor were any endangered or protected species sampled. No animals were handled during the survey, so no IACUC permit (a permit that is required to ensure the humane treatment and handling of captured animals) was required.

In the spring of 2007, an aerial line transect survey for black bears was conducted in the 21,398 km^2^ Skwetna study area in South-Central Alaska. This area is bordered on the south by the Chakachamna Lake, Chakachamna River and Cook Inlet; on the east by the Susitna and Yentna rivers; and on the north and west by the spine of the Alaska Range. Elevation ranged from 0 to 6,196 meters. Prominent habitats consisted of: birch and mixed birch-spruce forests, and muskegs at lower elevations; mixtures of grasslands and alder on the lower mountain slopes; alpine habitat at intermediate elevations; and rocky terrain with variable snow cover and glaciers at higher elevations. The survey was conducted between 10–25 May, a period after black bear den emergence and prior to full leaf-out of the birch forest.

**Fig 1:**
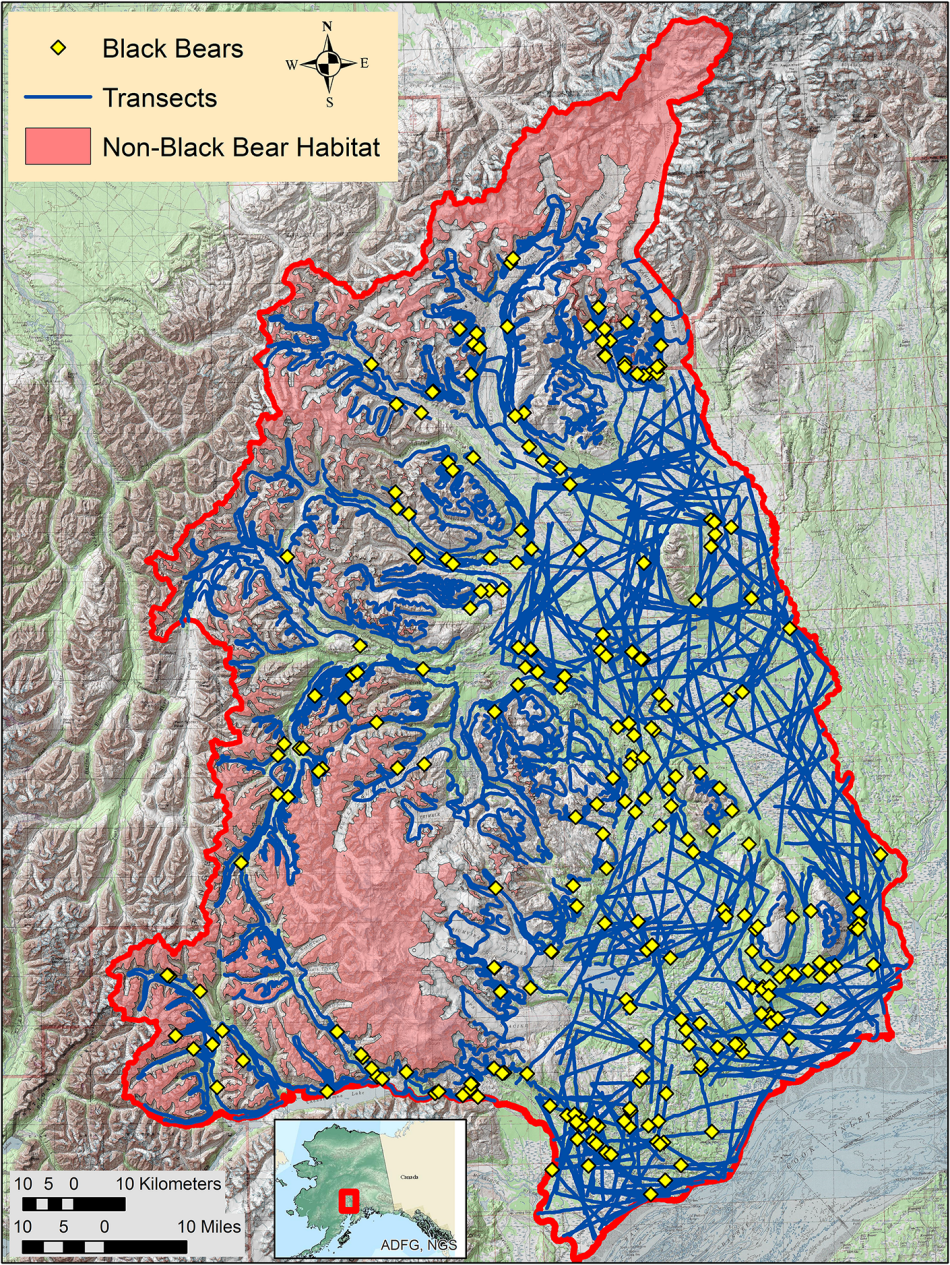
The Skwetna study area of South-Central Alaska that was used in a 2007 aerial distance sampling survey of black bears. The study area is enclosed by the red border and Cook Inlet to the southeast. Red-shaded areas within the study area denote locations that were above 1,067 m in elevation that were classified as non-black bear habitat. Yellow triangles denote the location of detected black bear groups. The blue lines denote the actual flown transects, with contour transects flown in mountainous terrain and straight transects that contain a midpoint hinge being flown in the lower-elevation, flat terrain.

A total of 470 30-km long transects were flown by 5 small tandem-seat aircraft that contained 2 observers: the pilot and a backseat observer. No black bears were encountered above 1,067 meters and terrain above this elevation was not considered black bear habitat. The 14,895 km^2^ of black bear habitat were sampled by 441 of the 470 flown transects. Roughly half of the bear habitat was too mountainous for straight-line transects and required contour transects [19–20]. Straight-line transects were hinged in the middle with a random angle between 30 and 330 degrees, and this facilitated efficient sampling of irregular shaped subareas that were suitable for “straight-line” transects (Fig 1). Bear searches were restricted to one-side of the transect to facilitate the collection of double-count data. Searches along contour transects were conducted on the uphill side, while a random side was used for straight-line transects. Other than using hinged straight-line transects, survey methodology follows the details given in Becker and Quang [20]. Possible covariates included: bear activity (bedded, sitting, standing, moving), group size, percent cover within 10 m of the bear, percent snow cover within 10 m of the bear, plane speed, search distance, and transect type (straight, contour). Percent cover and percent snow covariates were divided by 20, and air speed was divided by 100 to increase numerical efficiency of the optimizer used to obtain maximum likelihood estimates. Search distance represented how far out the observers were searching at the time the bear was detected and was measured by overflying that location and obtaining a GPS location, from which a distance to the transect was calculated. The covariate Bin.lnSD350 represents the search distance covariate which was truncated at *w*, transformed to the natural log scale, and binned to into 9 classes. The truncation and binning was done to minimize the correlation between this covariate and distance.

**Fig 2:**
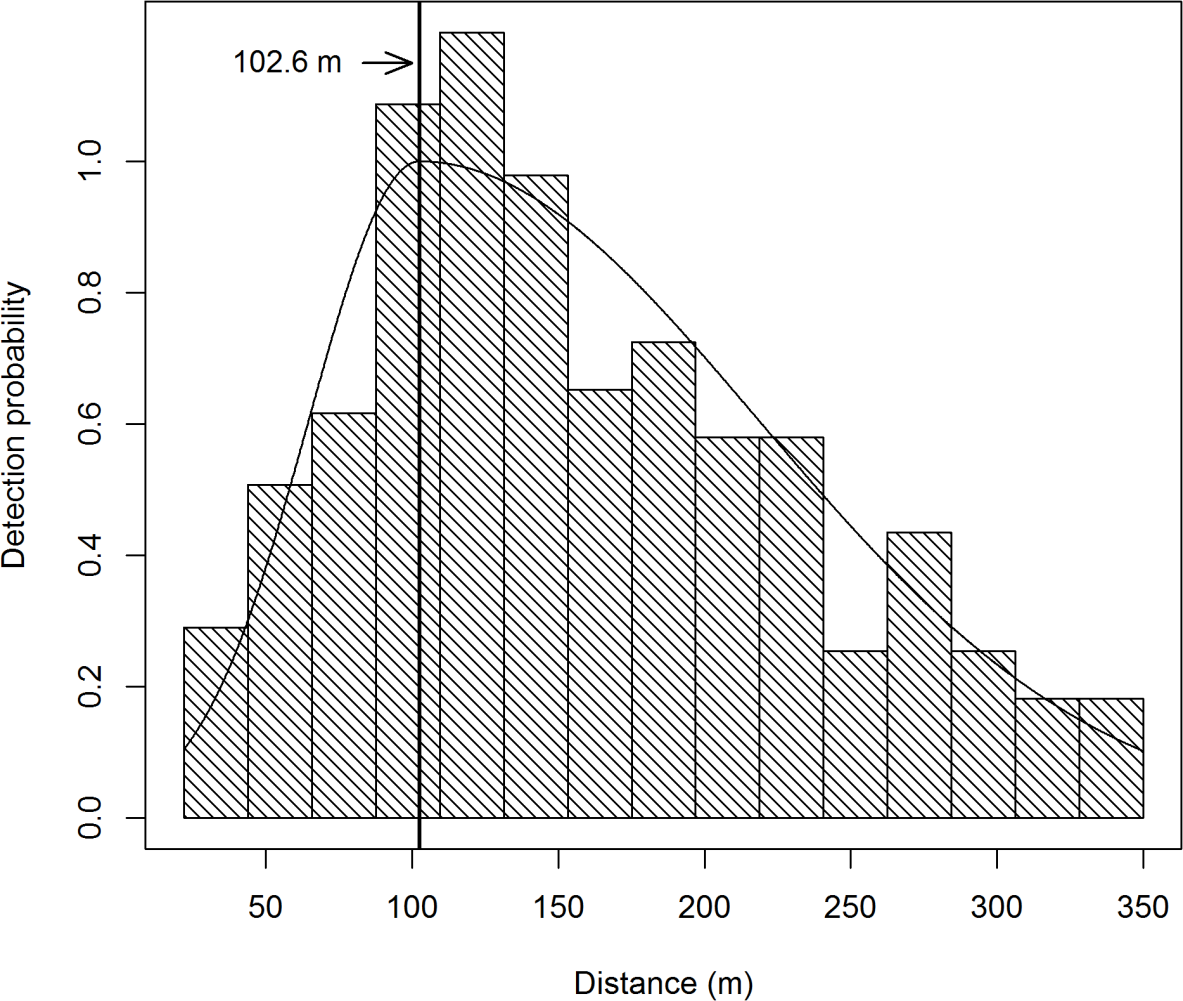
Two-piece normal detection model for black bears in Skwetna study area of South-Central Alaska. The detection curve is for averaged covariate values of binned search distance, percent cover, and transect type. The vertical line at 102.6 m depicts $\hat{\mu }$. The histogram is of the observed black bear group detection distances.

**Fig 3:**
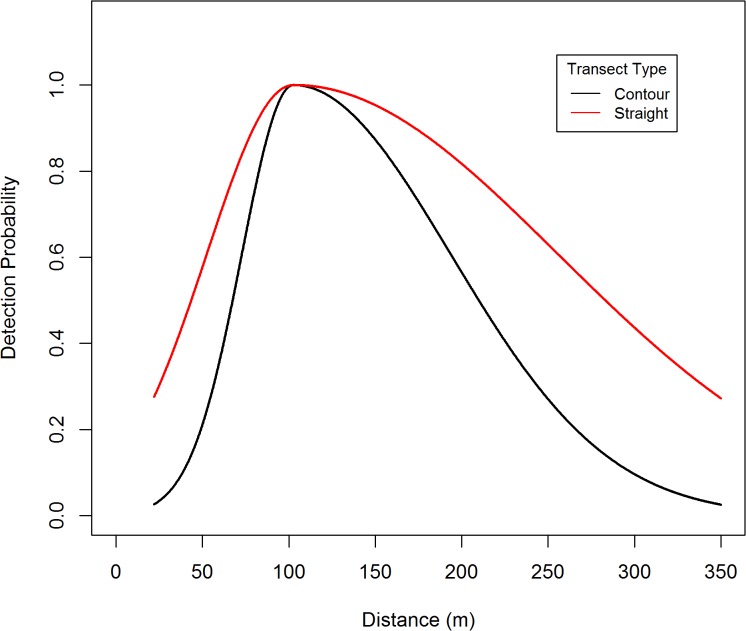
The effect of transect type on average covariate detection of black bears in the Skwetna study area of South-Central Alaska.

**Fig 4:**
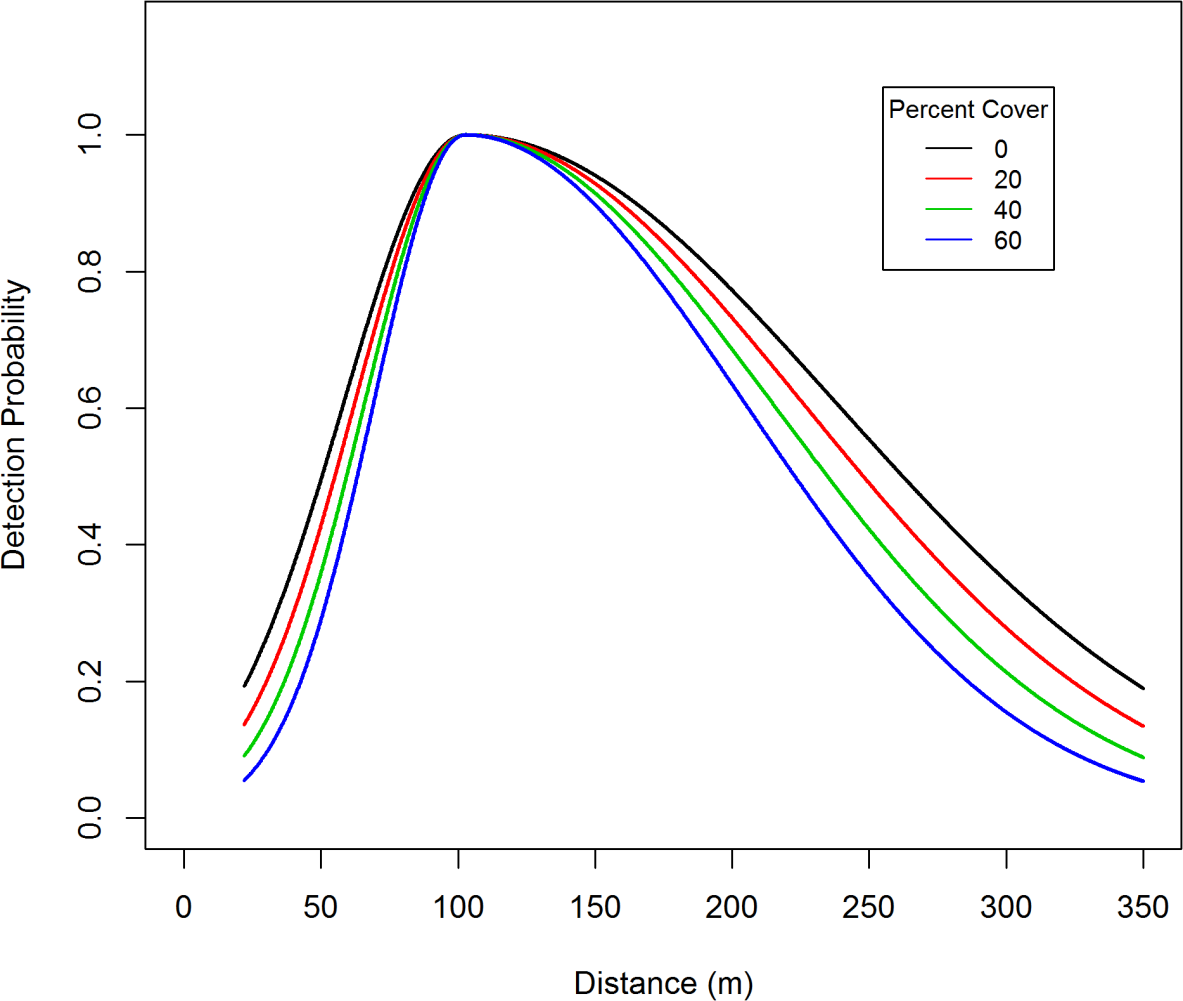
The effect of vegetative cover on average covariate detection of black bears in the Skwetna study area of South-Central Alaska.

**Fig 5:**
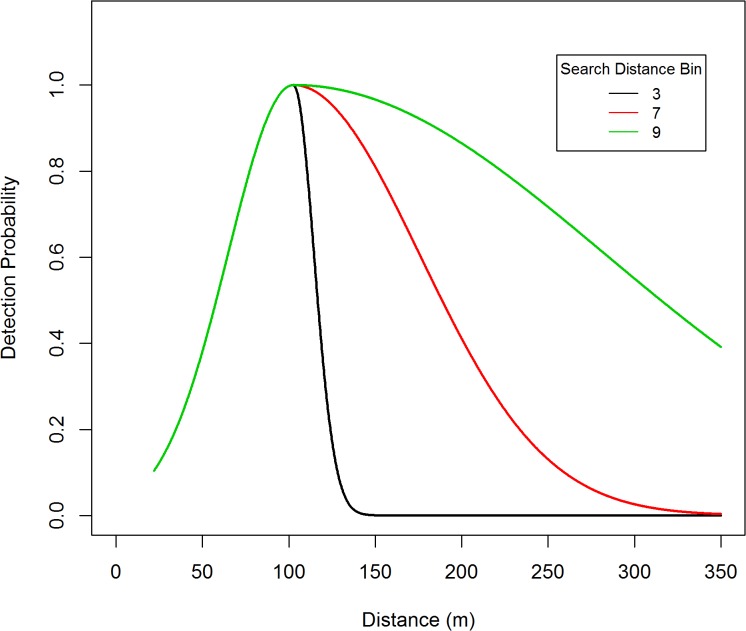
The effect of binned search distance on average covariate detection of black bears in the Skwetna study area of South-Central Alaska.

**Fig 6:**
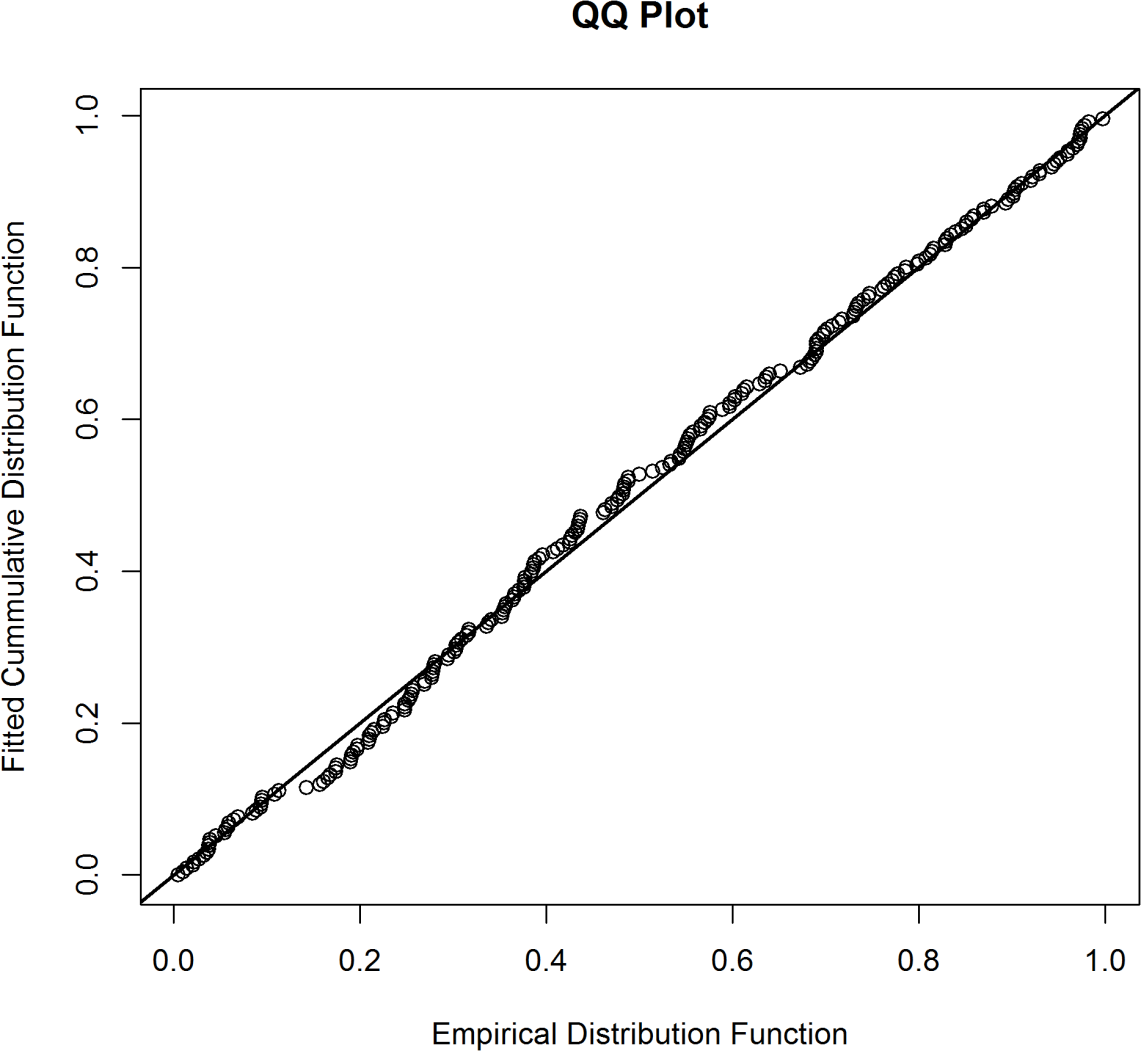
Q-q plot of the multiple covariate distance model fit.

A total of 260 black bear groups were detected from these transects. The distance data was truncated at 350 m (*w* = 350), in addition there was a 22 m blind spot underneath the plane, so $\pi \left(y\right)=\frac{1}{\left(350-22\right)}$. Within this 22–350 m strip, a total of 235 bear groups were detected, so 9.6% of the data were trimmed off. The distance data for these detections exhibited a unimodal distribution and were modeled using a two-piece normal detection function. The R-code [26] used to obtain the parameter estimates, standard errors, confidence intervals, graphs, and fit diagnostics are listed in appendix 1. Standard errors and confidence intervals were based on Borchers et al. [12] variance formula. Based on AIC, the best MCDS model (Fig 2) included covariates for transect type (Fig 3) and percent cover (Fig 4) which affect both scale parameters (*σ*_1*i*_,*σ*_2*i*_). The Bin.lnSD350 covariate was included in modeling the *σ*_2*i*_ scale parameter (Fig 5). The mode $\left(\hat{\mu }\right)$ was estimated to be 102.61 meters (se = 1.013). This analysis ignored observer type (pilot or backseat observer) which resulted in a detection model for the survey plane. Parameter estimates and standard errors are given in Table 1. A Goodness of Fit test for the MCDS model using 15 bins resulted in a Chi-sq. statistic of 7.742, df = 8, p = 0.459. A Kolmogorov-Smirnov test (p = 0.843) and a q-q plot (Fig 6) revealed no abnormalities. Collectively, these results indicate that the two-piece normal detection model provided an adequate fit to the data.

**Table 1:** Coefficients and standard errors for the best fitting multiple covariate distance sampling model using a two-piece normal detection model. The parameter notation Bin.lnSD denotes the 9 binned values of the natural log of truncated search distance (SD), for which ln(350) m was substituted for ln(SD) if the SD value was beyond 350 m (*w*).

Parameter	Coefficient	SE
Intercept	3.5549	0.0644
I(y> $\hat{\mu }$)	-2.5125	0.6103
I(y> $\hat{\mu }$) Bin.lnSD350	0.4526	0.0060
Transect (Flat)	0.5199	0.0358
Percent Cover	-0.0944	0.0044
ln($\hat{\mu }$)	4.6309	0.0128

**Fig 7:**
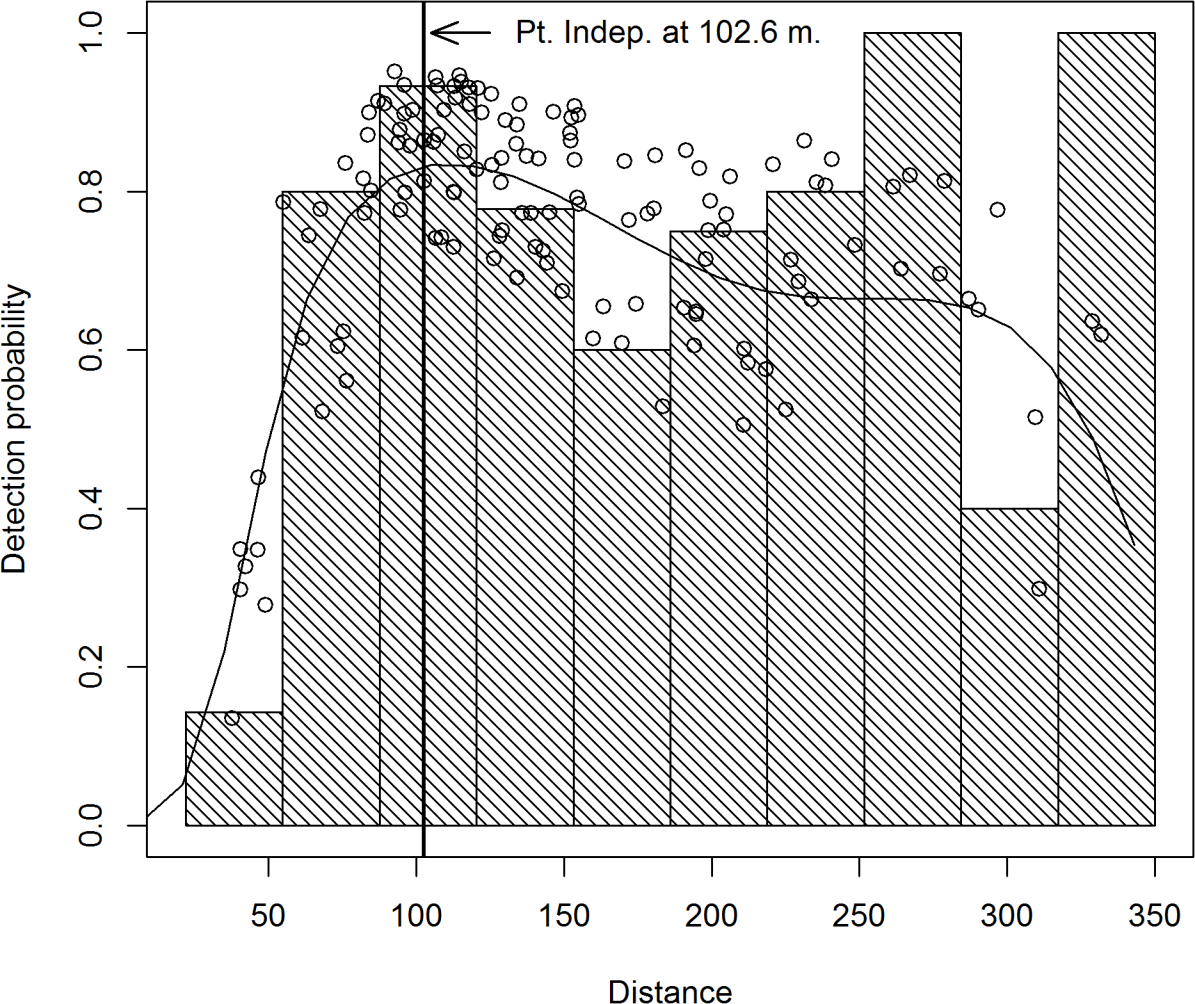
Logistic model of detection probabilities of the pilot given the backseat observer detected a black bear. The solid line denotes the conditional detection probability for the average values of the distance, percent cover, observer, air speed, and bedded/sitting bear covariates. The circles represent the model estimated conditional probabilities for each bear, based on their observed covariates.

**Fig 8:**
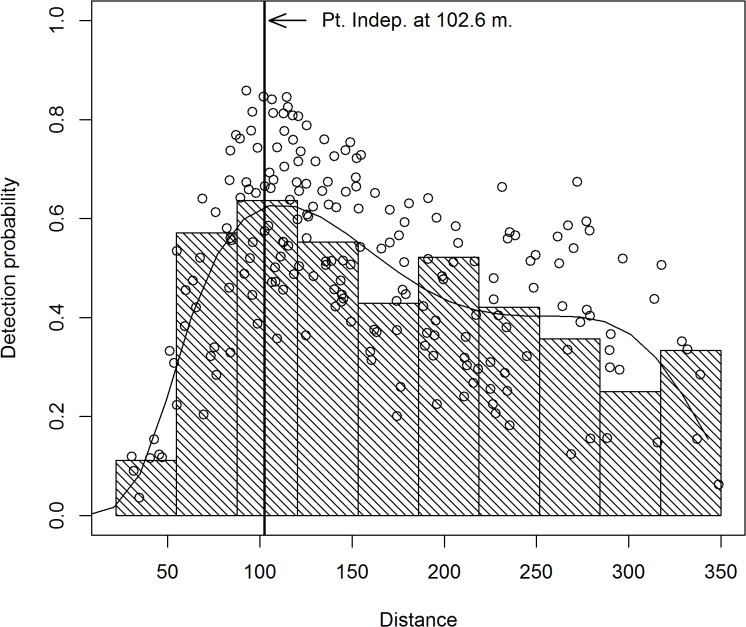
Logistic model of detection probabilities of the backseat observer given the pilot detected a black bear. The solid line denotes the conditional detection probability for the average values of the distance, percent cover, observer, air speed, and bedded/sitting bear covariates. The circles represent the model estimated conditional probabilities for each bear, based on their observed covariates.

The Double-Observer model was fit using the R-package mrds [26, 27]. In addition to the fixed observer effect, the covariates that were considered in the MCDS model for this data were also considered here. Based on AIC [28] (AIC = 3064.3), the best Double-Observer model included a 4^th^ order polynomial spline of distance, percent cover, air speed, bear activity of bedded/sitting, and observer. Parameter estimates are given in Table 2 and a graph of the average covariate values are given in Figs 7 and 8 for the pilot and backseat observer models respectively. A Goodness of Fit test resulted in a Chi-sq. value of 14.776, df = 11, p = 0.193. At 102.61 m ($\hat{\mu }$), the model resulted in an estimate of 0.926 (se = 0.038) for ${\hat{p}}_{•}\left(\hat{\mu }\right)$.

**Table 2:** Coefficients and standard errors for the best fitting double observer model which included a quadratic spline over distance. The 4-bs(distance, degree = 4) parameters denote the 4 parameters of the 4^th^ order polynomial spline of distance.

Parameter	Coefficient	SE
Intercept	2.2843	1.6794
bs(distance, degree = 4)1	9.1280	3.2812
bs(distance, degree = 4)2	-2.4780	2.7692
bs(distance, degree = 4)3	5.7301	3.5575
bs(distance, degree = 4)4	0.8770	1.6418
Observer (backseat)	-1.1690	0.2024
Percent Cover	-0.3819	0.1356
Air Speed	-2.8225	1.5882
Bedded/Sitting	-0.5289	0.2960

**Fig 9:**
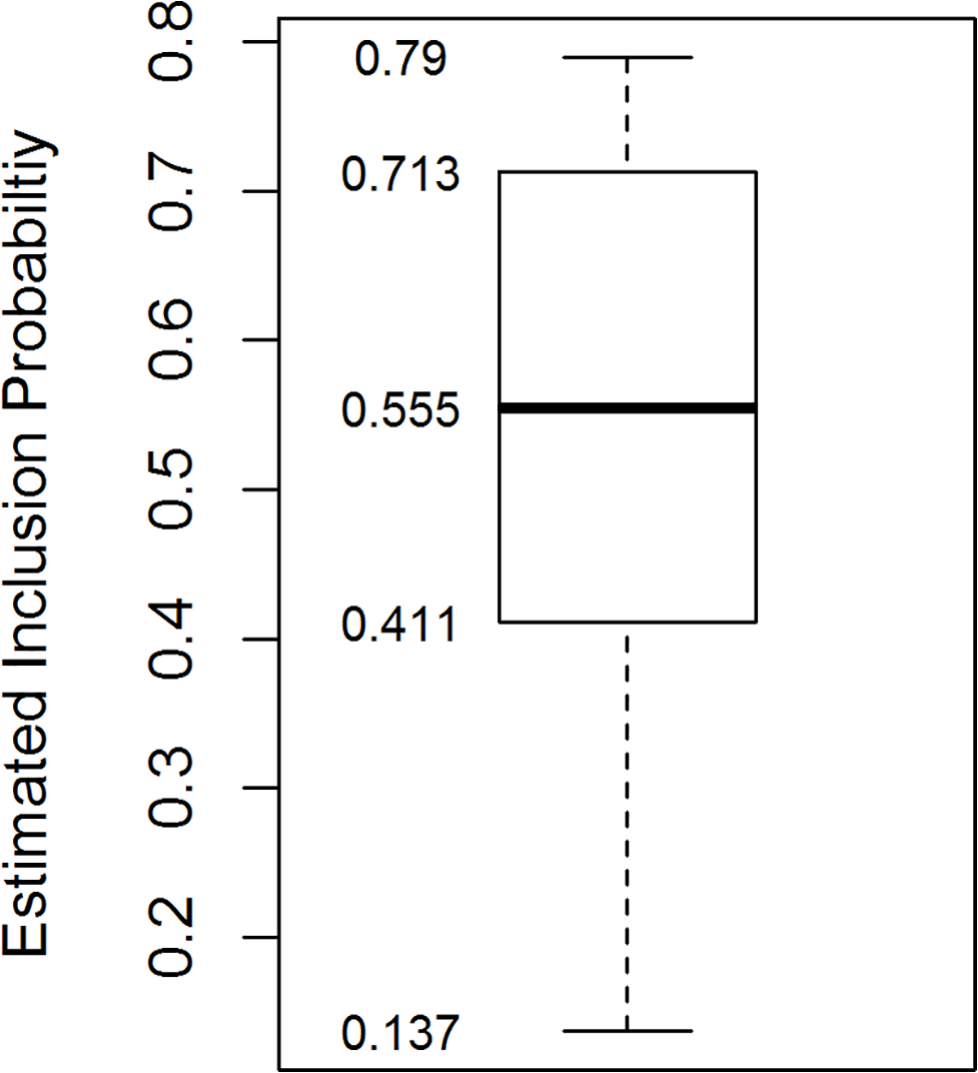
Boxplot of the estimated Horvitz-Thompson inclusion probabilities.

The estimated inclusion probabilities from our best MRDS model ranged from 0.137 to 0.790, with a median value of 0.555 (Fig 9). Only 2.98% of the estimated probabilities were below 0.2, and all were within the limits suggested by Marques and Buckland [5]. Our MRDS model resulted in a population estimate of 2,377.0 black bears (se = 185.29, CV = 7.80%) and a 95% confidence interval of (1990.9, 2838.1) in the study area. Converting to density (bears/1000 km^2^) results in a point estimate of 159.58 (se = 12.44) with a 95% confidence interval of (133.7, 190.5).

## Discussion

In our best MRDS model of the black bear dataset, distance was an important covariate and was fit using a 4^th^ degree polynomial spline. Our model did not restrict the apex locations of the MCDS and MRDS models to be the same. The apexes, 102.6 and 110.8 m respectively, differed by 8.2 meters. The main reasons the apexes were not restricted to be the same were: 1) distance may not be included in the best MRDS model, 2) computational complexity, and 3) if distance were in the MRDS model, unmodeled heterogeneity in the mark-resight data may cause the 2 apex locations to differ. In our black bear example, the difference in the estimates of ${\hat{p}}_{•}\left(\hat{\mu }\right)$ is negligible between these apex locations (0.926 and 0.928 respectively).

Besides goodness of fit tests to assess model fit, we also required the estimated inclusion probabilities obtained from the model to meet the recommendations of Marques and Buckland [5]. This additional criterion ensures that the population estimate is not being driven by a few observations with very low estimated inclusion probabilities. An analogous situation exists for traditional Horvitz-Thompson estimators [29] (where the inclusion probabilities for the observations are known, not estimated). The inclusion probabilities are contained in the denominator for both the point estimate and the variance. If very small inclusion probabilities are encountered then the variance becomes very large and the point estimate is driven by these few probabilities. In traditional sampling, care is taken to avoid small inclusion probabilities, and optimal sample designs have the inclusion probabilities proportional to size [29]. The recommendation of Marques and Buckland [5] formalizes the avoidance of this problem with their criteria of no estimated inclusion probabilities lower than 0.1, and less than 5% falling in the 0.1 to 0.2 range.

Our black bear example illustrates the utility of distance sampling to make inferences over large study areas. Areas of management interest are often so large that aerial distance sampling is one of the few logistically feasible options. Double-count modeling is needed since the assumption of perfect detection at the detection apex would not be tenable without supporting data. Our double count model indicates that perfect detection at the apex may be reasonable for some black bear sightings but not for all sightings. This is consistent with our expectations that black bears are not perfectly detectable at some apex distance from fast-moving aircraft over various covariate conditions. A MCDS estimate that assumes perfect detection at the apex would generate a population estimate of 2,220.6 bears while a MRDS model assuming full independence would estimate 1,716.6 bears. These estimates are 7.4% and 27.8% lower than our reported estimate of 2,377.0 black bears.

Very few options exist for modeling distance sampling data that exhibit a unimodal form. The gamma detection model developed by Becker and Quang [20] allows for covariates and has proven to be a useful model with aerially collected distance sampling data if the assumption of perfect detection at the apex is reasonable. To determine if that assumption is reasonable, double-observer data must be collected [14]. If the analysis of the double-count data indicates a MRDS model is required, the use of the gamma detection function to model the distance data could be problematic. Using a gamma detection function in a MRDS model with PI would require a single apex location for the distance model, so no covariates could be used; thus a CDS model using a gamma detection function would model the distance data. When appreciable variation in animal detection exists, as in the black bear example, the inability of the CDS model to incorporate covariates would result in an inferior estimate (AIC = 3111.5 versus 3064.3 for our model) [5]. Neilson et al. [30] used a non-monotonic, non-parametric Gaussian kernel estimator to model aerially collected distance data on golden eagles (*Aquila chrysaetos*) with a MRDS model that used the PI assumption. Their nonparametric detection model is more flexible than a unimodal detection model, but does not allow for covariates in the distance portion of the MRDS model.

The purpose of this paper is to model unimodal distance datasets with a MRDS model that addresses unmodeled heterogeneity in the mark-resight data and allows for the use of covariates in modeling animal detection. There are several approaches that could be modified to provide a solution. The limited independence model 17] is one possible solution; the logistic detection function may have to be modified to adequately model the unimodal aerial distance data. The probit-Bernoulli model [18] is another possible solution; it would need to be modified to model observer dependence as a function of distance from the detection apex. This model may allow for multiple detection apexes. We choose to take the approach of Borchers et al. [12] to solve this problem. We used the two-piece normal detection function with covariates in a MRDS model with the PI assumption obtain a population estimate from our aerially collected bear data. This approach provided a good fit to our unimodal detection data, avoids the unrealistic assumption of perfect detection at the apex, avoids the FI assumption that has caused biases in many MRDS datasets, and allows for the use of covariates in the MCDS model to obtain a potentially superior model [5]. Researchers with unimodal detection data should consider the two-piece normal detection model for MRDS models and MCDS models.

## Supporting Information

S1 File BlackBear.MRDS.data.csv. A csv file containing the black bear distance data that was analyzed in the manuscript; this file is required by PlosOneMRDSAnalysis.txt.
(CSV)

S2 File SkwetnaTranData350.csv. A csv file containing the transect area information; this file is required by SkwetnaBlackBearAnalysis.txt.
(CSV)

S1 Text PlosOneMRDSAnalysis.txt. A R script file that will replicate the results found in this manuscript.
(TXT)

S2 Text Two.Piece.Normal.txt. A R file containing all the R-functions written to fit a MRDS model that uses a two-piece normal detection model. These functions are required by the SkwetnaBlackBearAnalysis.txt file.
(TXT)
