# Correction: A Unimodal Model for Double Observer Distance Sampling Surveys

**DOI:** 10.1371/journal.pone.0140913

**Published:** 2015-10-14

**Authors:** Earl F. Becker, Aaron M. Christ

There are errors in S1 Text and S2 Text that affect the code script. The corrected S1 Text fixes an error in creating Fig. 9. The corrected S2 Text fixes an error that masked the location of a needed function. Please find the corrected files here.

## Supporting Information

S1 Text PlosOneMRDSAnalysis.txt. A R script file that will replicate the results found in this manuscript.
(TXT)

S2 Text Two.Piece.Normal.txt. A R file containing all the R-functions written to fit a MRDS model that uses a two-piece normal detection model. These functions are required by the SkwetnaBlackBearAnalysis.txt file.
(TXT)
